# Accuracy of the Dynamic Acoustic Map in a Large City Generated by Fixed Monitoring Units

**DOI:** 10.3390/s20020412

**Published:** 2020-01-11

**Authors:** Roberto Benocci, Chiara Confalonieri, Hector Eduardo Roman, Fabio Angelini, Giovanni Zambon

**Affiliations:** 1 Department of Earth and Environmental Sciences (DISAT), University of Milano-Bicocca, Piazza della Scienza 1, 20126 Milano, Italy; c.confalonieri12@campus.unimib.it (C.C.); fabio.angelini@unimib.it (F.A.); giovanni.zambon@unimib.it (G.Z.); 2 Department of Physics “G. Occhialini”, University of Milano-Bicocca, Piazza della Scienza 3, 20126 Milano, Italy; eduardo.roman@mib.infn.it

**Keywords:** noise mapping, noise mitigation, DYNAMAP project

## Abstract

DYNAMAP, a European Life project, aims at giving a real image of the noise generated by vehicular traffic in urban areas developing a dynamic acoustic map based on a limited number of low-cost permanent noise monitoring stations. The system has been implemented in two pilot areas located in the agglomeration of Milan (Italy) and along the Motorway A90 (Rome-Italy). The paper reports the final assessment of the system installed in the pilot area of Milan. Traffic noise data collected by the monitoring stations, each one representative of a number of roads (groups) sharing similar characteristics (e.g., daily traffic flow), are used to build-up a “real-time” noise map. In particular, we focused on the results of the testing campaign (21 sites distributed over the pilot area and 24 h duration of each recording). It allowed evaluating the accuracy and reliability of the system by comparing the predicted noise level of DYNAMAP with field measurements in randomly selected sites. To this end, a statistical analysis has been implemented to determine the error associated with such prediction, and to optimize the system by developing a correction procedure aimed at keeping the error below some acceptable threshold. The steps and the results of this procedure are given in detail. It is shown that it is possible to describe a complex road network on the basis of a statistical approach, complemented by empirical data, within a threshold of 3 dB provided that the traffic flow model achieves a comparable accuracy within each single groups of roads in the network.

## 1. Introduction

Road traffic noise is one of the foremost problems in Europe, with more than 100 million people exposed to Lden (day-evening-night) levels higher than 55 dB (A) [1]. Consequently, scientific communities and authorities started observing the surge of noise-related health problems such as sleep disorders and tiredness associated with a long-term road traffic noise exposure [2,3], relationships between annoyance and exposure to transportation noise [4], increased cardiovascular risk and hypertension [4,5,6], mental performance [7], and students cognitive disorders [8,9].

The increasing awareness on these issues, promoted by EU policies through the Environmental Noise Directive (END) of 2002, its revision [10,11] and integrated approaches (CNOSSOS-EU) [12,13], encouraged the use of distributed monitoring systems and noise mapping in the control of noise exposure.

Mitigation measures in urban and near-urban contexts need to be identified according to a realistic picture of noise distribution over extended areas. This requirement demands for real-time measurements and processing to assess the acoustic impact of noise sources. In this framework, noise maps might represent an important tool. They are based on collecting and processing information on the traffic flow averaged over long periods of time [14] using acoustic models [15] rather than unattended phono-metric measurements, which, on the other hand, are typically used to validate results from computational models [16,17].

Recently, the development of dynamic noise maps is gaining interest because of the realistic soundscape picture they can provide in complex traffic network. Different approaches have been pursued, motivated by the fact that noise fluctuations might be important to evaluate sleep disturbance and noise annoyance [18,19]. Other recent approaches regard participatory sensing, which enables any person to take measurements using either specific measurement equipment or mobile phones [20,21]. In addition, mobile sampling could, in principle, increase temporal and spatial resolution, even with short length samples [22], in a more controlled environment than participatory sensing, since the measurement is carried out by trained people. A usual practice, which integrates traditional noise mapping and participatory sensing, is to take on-site measurements to calibrate the noise map based on computational models or to use them to dynamically update noise maps based on interpolation schemes [23,24].

Noise mapping recently moved towards a multi-source approach [25,26]. Specifically, advanced probabilistic noise modelling based on source-oriented sound maps within an open-source Geographic Information System (GIS) environment allows the production of traffic, fountains, voices and birds sound maps and to investigate the competition between sound sources [27].

In this continuously evolving scenario, DYNAMAP, a co-financed project by the European Commission through the Life+ 2013 program, started its activities in 2014 [28]. It aimed at developing a dynamical acoustic map in two pilot areas: a large portion of the urban area of the city of Milan (District 9) and the motorway surrounding Rome. In both cases, we developed a method for predicting the traffic noise in an extended area using a limited number of monitoring sensors and the knowledge of traffic flows.

The development of automatic noise mapping systems delivering short-term noise maps (dynamic noise maps) are not explicitly required by the END. However, their automatic generation is estimated to reduce the cost of long-term noise assessment by 50%, adding significant benefits for noise managers and the public through updated information and dedicated web tools with the opportunity to control noise with alternative measures based on traffic control and management. While this approach seems quite promising in purely suburban areas, where noise sources are well identified, in complex urban scenarios further considerations are needed.

Regarding a suburban area, a detailed study has been performed for the motorway zone around the city of Rome. The pilot area of Rome is located along a six-lane ring road (A90) surrounding the city, going through many suburban areas where the presence of single or multiple noise sources, such as railways, crossing, and parallel roads, impact the residents. Pre-calculated basic noise maps, prepared for different sources, traffic, and weather conditions, are updated from the information retrieved from 19 distributed noise sensors. Difficulties lied in the contribution of multiple noise sources and in the influence of meteorological conditions when receptors are located at a distance from the road greater than 80 m. The final assessment on DYNAMAP reliability and accuracy in the suburban area of Rome can be found in [29].

For an urban environment, we review in this paper the case of Milan, where DYNAMAP has been implemented in a pilot area, namely District 9, consisting of about 2000 road arches in the north-east part of the city. Due to the high number of potential noise sources needed to be monitored, we decided to adopt a statistically based approach. This is the outcome of previous investigations [30], proving that the noise emission from a street generally depends on its use and activity in the urban context, therefore suggesting a stratified sampling aimed at optimizing the number of monitoring sites.

In both scenarios, being urban or suburban, the presence of anomalous noise events (ANEs), that is events that are extraneous to the actual vehicle noise, may alter the noise levels represented by DYNAMAP. For this reason, dedicated algorithms have been implemented in ARM-based (see Acronyms Sect. for definition) acoustic sensors showing the feasibility of the method both in terms of computational cost and classification performance [31] with the purpose of identifying and removing ANEs from the time series, thus restricting the acoustic data to the traffic source only [32]. In particular, different typologies of anomalous noise events have been described statistically and associated with the identified street clusters of the city of Milan [33]. Similar approaches based on permanent monitoring network and street categorization are now adopted in other cities [34].

In this paper, we provide a review of DYNAMAP project in the pilot area of the city of Milan. It represents the final assessment on its accuracy and reliability obtained from the comparison between field measurements and map predictions.

## 2. Materials and Methods

**Figure 1:**
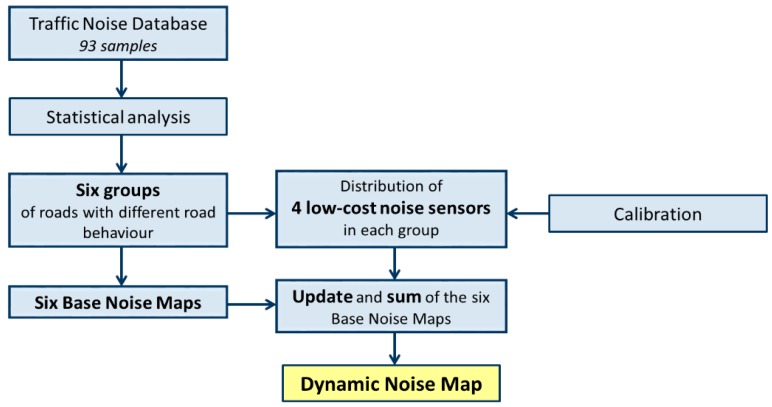
General block diagram of the followed processes in DYNAMAP.

In this section, we provide an overview of the general scheme of DYNAMAP implemented in Milan, including the initial sampling campaign, the statistical analysis, the calculation and map updating procedure, and the methodology for the system validation (calibration of sensors, their reliability, and field measurements). Figure 1 shows a general block diagram of the following processes for illustrative purposes.

### 2.1. Initial Sampling Campaign and Statistical Analysis

A database of noise time series belonging to the road network of Milan was necessary to characterize the traffic noise of the city. For this reason, 93 traffic noise recordings of 24 h represented our initial large-scale noise monitoring investigation [35]. Given the large number of roads in the city of Milan, in order to determine the acoustic behavior of different roads we applied a statistical approach based on a cluster analysis. The open source software “R” [36] was applied for clustering and the package “clValid” [37,38] was used for validating the results of the different cluster algorithms. The ranking provided by the “clValid” R-package showed the best performance of the hierarchical clustering with Ward algorithm [39], as detailed in [40].

**Figure 2:**
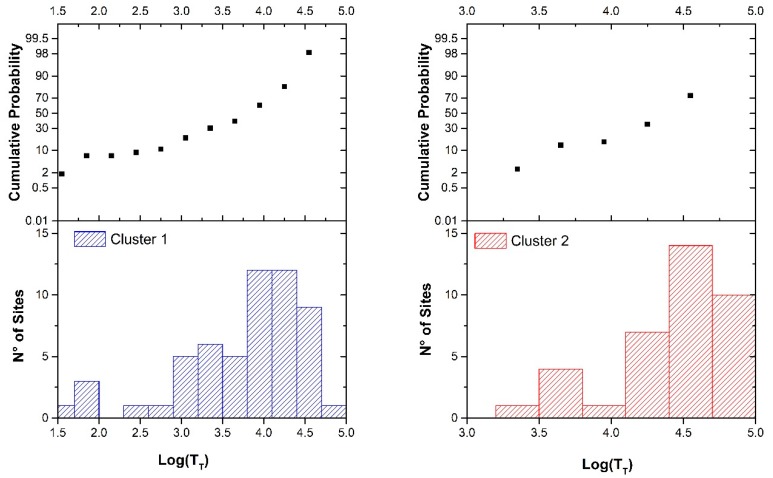
Number of sites and cumulative probability for Cluster 1 (left side) and Cluster 2 (right side) as a function of the non-acoustic parameter *x* = Log(T_T_). Bin size is 0.3.

The results showed two main noise behaviors correlated to vehicle flow patterns [41,42]. Its extension to non-monitored roads needed an available non-acoustic road-related parameter [43] and we found the logarithm of the total daily traffic flow, Log(T_T_) to be a convenient quantity. The number of events and the cumulative probability for the resulting two clusters, as a function of the non-acoustic parameter *x* = Log(T_T_), are illustrated in Figure 2.

As we are interested in finding an analytical representation for the distribution functions, P(x), in each cluster, we studied the corresponding cumulative distributions of *x*, *I(x)*, which have been fitted using an analytical expression:

(1) $$I\left(x\right)=10^{f\left(x\right)}=\int_{0}^{x}dy\;P\left(y\right);\;\mathrm{with}\;\underset{x\to \infty }{\lim}I\left(x\right)=1.$$

Deriving I(x), we get

(2) $$\frac{dI\left(x\right)}{dx}=P\left(x\right)-P\left(0\right).$$

**Figure 3:**
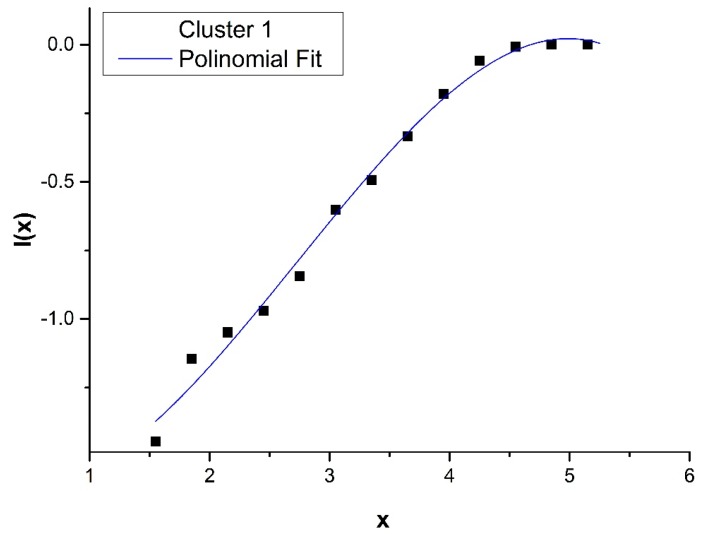
Cumulative distribution *I(x)* for Cluster 1 fitted using the analytical expression, Equation (1), *I(x)* = 10*^f(x)^,* where f(x) is a polynomial of third degree and x = Log(T_T_).

**Figure 4:**
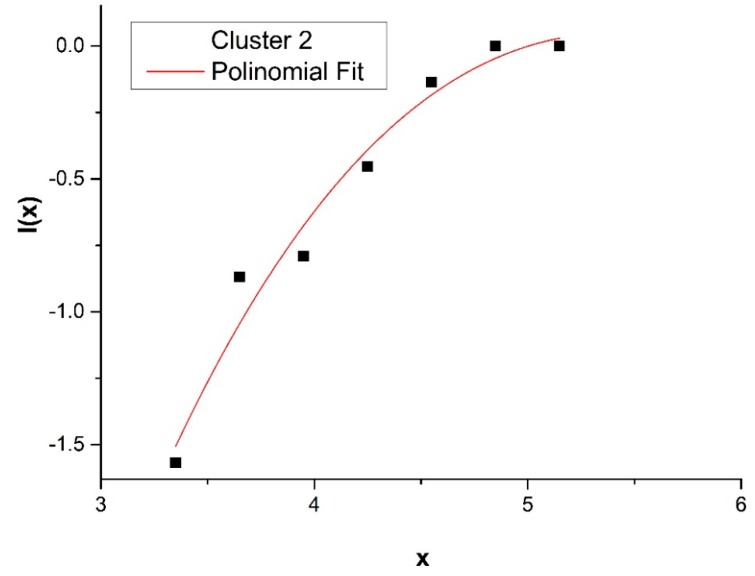
Cumulative distribution *I(x)* for Cluster 2 fitted using the analytical expression, Equation (1), *I(x)* = 10*^f(x)^*, where f(x) is a polynomial of third degree and x = Log(T_T_).

The probability distribution *P(x)* can be obtained from the analytical fit of the cumulative distribution *I(x)* according to the relation:

(3) $$P\left(x\right)=\ln\left(10\right)f^{\prime }\left(x\right)I\left(x\right),$$

where f(x) is a polynomial of third degree and $f{}^{\prime }\left(x\right)$ is the derivative of f(x). The results of I(x) for Clusters 1 and 2 are reported in Figure 3 and Figure 4.

Analytical fit functions $f_{1}$ and $f_{2}$ for P(x) for the two clusters are:

(4) $$f_{1}\left(x\right)\;=\;-1.55545\;-\;0.24459\;x\;+\;0.28834\;x^{2}\;-\;0.03526\;x^{3}$$

(5) $$f_{2}\left(x\right)\;=\;-15.21817\;+\;7.01263\;x\;-\;1.02922\;x^{2}\;+\;0.04708\;x^{3}$$

**Figure 5:**
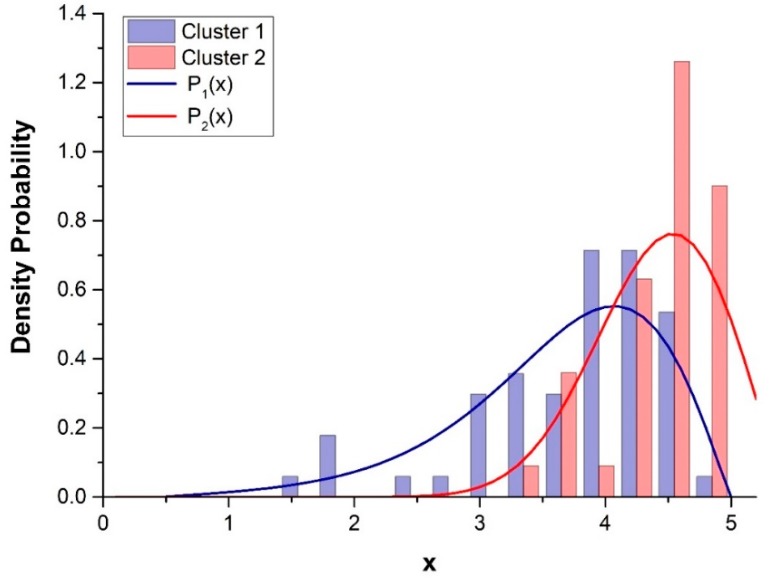
Distribution functions *P*_1_*(x)* and *P*_2_*(x)* (Equations (3)–(5)) for Clusters 1 and 2; *x* = Log (T_T_)_._

In Figure 5, the histograms and density function, *P*_1_*(x)* and *P*_2_*(x)* for Cluster 1 and 2 (for the initial 93 sample measurements) are illustrated as a function of the non-acoustic parameter, *x*. Here, *P*_1_*(x)* and *P*_2_*(x)* represent the “probability” that a road with a given *x* belongs to Clusters 1 and 2, respectively.

In general, owing to the large superposition of the two cluster distributions *P*_1_*(x)* and *P*_2_*(x),* we might consider a linear combination between the two mean normalized cluster profiles to describe the noise behavior of a road with a given value of *x*.

The weights (α_1_, α_2_) of the linear combination can be obtained, for each value of *x*, using the relations: α_1_ = *P*_1_*(x)* and α_2_ = *P*_2_*(x)*. Therefore, the values of α_1,2_ represent the probability that a given road characterized by its own value of *x* belongs to the corresponding Clusters, 1 and 2. By denoting as β, the normalized values of α_1,2_, we obtain:

(6) $$\begin{array}{c} \beta_{1}\;=\frac{\alpha_{1}}{\;\alpha_{1}+\alpha_{2}}\\ \beta_{2}\;=\frac{\alpha_{2}}{\alpha_{1}+\alpha_{2}} \end{array}$$

For practical use, we cannot describe the behavior of each single road in the network, therefore, the entire range of variability of the non-acoustic parameter has been divided into six intervals in such a way that each group contains approximately the same number of roads. In this way, all road stretches within a group are represented by the same acoustic map, while six groups are found to be suitable for our purposes. The noise in a given location will be predicted by a combination of the six acoustic base noise maps whose variation (dynamic feature) is provided by field stations. The process for updating the pre-calculated six base noise maps is based on the average of noise level variations recorded by the monitoring stations, according to two different procedures described below.

### 2.2. Dynamic Map

For the actual implementation of DYNAMAP, we relied on 24 monitoring stations that have been installed homogeneously in the six groups *g* (four in each group), in such a way to reproduce the empirical distribution of the non-acoustic parameter in District 9 (to be noted that the 24 fixed monitoring units have been installed in sites belonging to the pilot area and not corresponding to the locations where the 93 sample measurements have been recorded).

The noise signal from each station j is filtered from any anomalous events not belonging to road traffic noise prior to its integration to obtain *Leq^τ^_j_* over a predefined temporal interval τ (τ = 5, 15, 60 min) [32,33,34]. Thus, we get 24 *Leq^τ^_g,j_* values every τ min, each one corresponding to a recording station *j* and belonging to a group *g*. To update the acoustic maps, we deal with variations, $\delta_{g,j}^{\tau }\left(t\right)$, where the time t is discretized as *t* = nτ and n is an integer, defined according to:

(7) $$\delta_{g,j}^{\tau }\left(t\right)=Leq_{g,j}^{\tau }\left(t\right)\;-\;Leq_{ref\;g,j}^{}\left(T_{ref}\right)$$

where *Leq_ref g,j_*$\;\left(T_{ref}\right)$ is a reference value calculated from the acoustic map of group *g* (using CADNA model) at the time interval *T_ref_* = (08:00–09:00) at the point corresponding to the position of the (*g, j)-*th station. The CADNA software provides mean hourly *Leq* values over the entire city of Milan at a resolution of 10 m given a set of input traffic flow data, thus representing a reference static acoustic map, *Leq_ref g,j_* $\left(T_{ref}\right)$. Here, we have chosen the reference time *T_ref_* = (08:00–09:00) for convenience, since it displays rush-hour type of behavior. The predefined temporal ranges within the day are:

τ = 5 min for (07:00–21:00); τ = 15 min for (21:00–01:00); τ = 60 min for (01:00–07:00).

This choice has been motivated by the need to provide the shortest time interval for the update of the acoustic maps keeping the associated error approximately constant over the entire day [44].

### 2.3. Average Over the Monitoring Stations in Each Group: 1st Method

In this section, we discuss how to use the 24 $\delta_{g,j}^{\tau }\left(t\right)$ defined in Equation (7) in such a way to bring DYNAMAP to operation. We used two methods: the first described in this section and the second in Section 2.4. The first method is quite straightforward and implies that once all the $\delta_{g,j}^{\tau }\left(t\right)$ values are provided, the six acoustic maps corresponding to each group *g* can be updated by averaging the variations in Equation (7) over the four monitoring station values j in each group, according to [43,45]:

(8) $$\delta_{g}^{\tau }\left(t\right)=\;\frac{1}{4}\Sigma_{j=1}^{4}\delta_{g,j}^{\tau }\left(t\right)$$

### 2.4. Clustering of the 24 Monitoring Stations: 2nd Method

**Figure 6:**
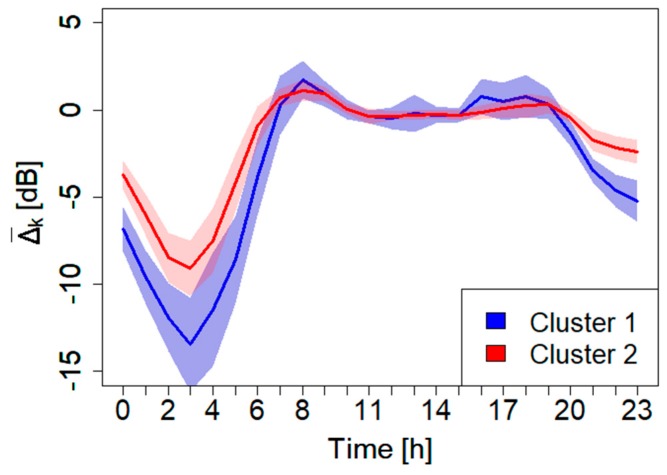
Mean normalized cluster profiles, ${\bar{\Delta }}_{k}$, and the corresponding error band, *k* indicates the cluster index. Time resolution τ= 60 min. The colored band represents the 1σ confidence level. In these calculations, the normalized noise level is obtained following the procedure described in [40].

**Figure 7:**
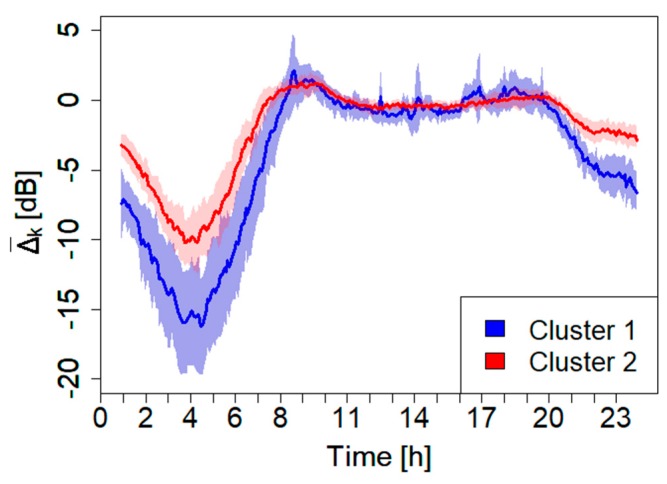
Mean normalized cluster profiles, $\;{\bar{\Delta }}_{k},$ and the corresponding error band, *k* indicates the cluster index. Time resolution τ= 5 min. The colored band represents the 1σ confidence level. The normalized level used here is the same as the one determined in Figure 6.

The second procedure for updating the acoustic maps is based on a two-cluster expansion scheme, which uses all the 24 stations to determine $\delta_{g}^{\tau }\left(t\right)$ simultaneously (see Section 2.6 for details on the stations network). The clustering method, as described in Section 2.1, is applied here to determine the two corresponding clusters. For this purpose, we used the 24 h noise profiles recorded by each monitoring sensor over the period from 13th November 2018 to 5th February 2019. From this ANE-free dataset, we excluded all festivities, weekends, rainy, and windy days. In order to get robust noise profiles, we manually calculated, for each sensor, its median. For this analysis, we chose two time resolutions, τ, constant for all the day: τ= 60 and 5 min. The results of the analysis, performed on the 24 median profiles, are reported in Figure 6 and Figure 7.

From this analysis, it appears very clearly the robustness of the clustering method of the 24 monitoring sensors (for both τ= 60 and 5 min). In fact, the 24 sensors result perfectly distributed in the two clusters mimicking the trend obtained with the original sampling measurements taken over the entire city. In Table 1, the information regarding the monitoring sensors together with their cluster membership are reported.

#### Updating Procedure for the 2nd Method

**Figure 8:**
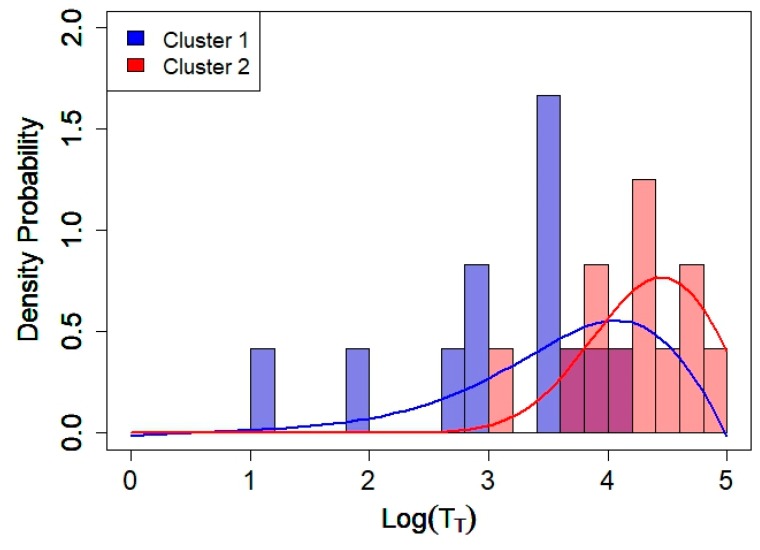
Histograms (from the 24 monitoring stations) and probability distributions, *P*_1_*(x)* and *P*_2_*(x),* as a function of the non-acoustic parameter, *x* = Log(T_T_), for Clusters 1 and 2. Bin size is 0.2. *P*_1_*(x)* and *P*_2_*(x)* are the same functions shown in Figure 5.

Once the compositions of Clusters 1 and 2 have been found (meaning that there are *N*_1_ stations in Cluster 1, *k*_1_ = (1, …, *N*_1_), and *N*_2_ stations in Cluster 2, *k*_2_ = (1, …, *N*_2_), such that *N*_1_ + *N*_2_ = 24), we need to rearrange the variations obtained from Equations (7) and (8) according to the indices *C*_1,*k*1_ and *C*_2,*k*2_, which we denote as $\delta_{C1,k1\;}^{\tau }\left(t\right)$ and $\delta_{C2,k2\;}^{\tau }\left(t\right)$ within each cluster, *C*_1_ and *C*_2_. Then, we calculate the mean variations, $\delta_{C1}^{\tau }\left(t\right)$ and $\delta_{C2}^{\tau }\left(t\right)$, for each cluster according to,

(9) $$\begin{array}{c} \delta_{C1}^{\tau }\left(t\right)=\frac{1}{N_{1}}\Sigma_{k1=1}^{N_{1}}\;\delta_{C1,k1}^{\tau }\left(t\right)\\ \delta_{C2}^{\tau }\left(t\right)=\frac{1}{N_{2}}\Sigma_{k2=1}^{N_{2}}\;\delta_{C2,k2}^{\tau }\left(t\right), \end{array}$$

where *C*_1_,*_k_*_1_ and *C*_2_,*_k_*_2_ are indices of stations belonging to Cluster 1 and Cluster 2, respectively. In Figure 8, the histograms of the non-acoustic parameter, *x* = Log(T_T_), for Clusters 1 and 2 of the 24 sensors (shown in Figure 6 and Figure 7) are illustrated. For comparison, the density function *P*_1_*(x)* and *P*_2_*(x)* obtained for the initial 93 sample noise time series (shown in Figure 5) are also included. The rather good agreement allows using such distribution functions to express the mean variation $\delta_{g}^{\tau }\left(t\right)$ associated with each group *g* using the formula:

(10) $$\delta_{g}^{\tau }\left(t\right)=\bar{\beta_{1}}\left({\bar{x}}_{g}\right)\;\delta_{C1}^{\tau }\left(t\right)+\;\bar{\beta_{2}}\left({\bar{x}}_{g}\right)\;\delta_{C2}^{\tau }\left(t\right)$$

Here, the value ${\bar{x}}_{g}$ represents the mean non-acoustic parameter associated with group *g*, and $\bar{\beta }$_1_(${\bar{x}}_{g}$), $\bar{\beta }$_2_(${\bar{x}}_{g}$) the corresponding probabilities to belong to Clusters 1 and 2, respectively (see Table 2 for the mean values of $\bar{\beta }$_1_ and $\bar{\beta }$_2_ for the six groups and Equation (6) for their definition).

### 2.5. Dynamic Noise Level at an Arbitrary Location

The absolute level *Leq^τ^* *_s_*(t) at an arbitrary site *s* at time *t* can be obtained from the measured values of $\delta_{g}^{\tau }\left(t\right)$ using either Equation (8) or Equation (10). The first quantity we need to know is the value of *Leq_ref g,s_* that is the reference *Leq* calculated in the point *s* at the reference time (8:00–9:00) due to group *g*, which is provided by CADNA model (acoustic base map). The absolute level *Leq^τ^* *_s_*(t) at location *s* at time *t = nτ* can then be obtained by combining the level contribution of each base map with its variation $\delta_{g}^{\tau }$(*t*):

(11) $$Leq_{s}^{\tau }\left(t\right)=10\;Log\;\overset{6}{\underset{g=1}{\sum }}10^{\frac{Leq_{ref_{g,s}\;}+\delta_{g}^{\tau }\left(t\right)}{10}}$$

This operation provides what we called the “scaled map” (dynamic map).

### 2.6. Measurement Campaign

A measurement campaign, completed in 2019, aimed at testing the results of DYNAMAP predictions. This has been justified by the updated release of anomalous noise events detection (ANED) algorithm which acts directly on the recorded noise time series from the 24 monitoring stations prior to their use in the DYNAMAP calculation process (see below). It presented a higher recognition efficiency of anomalous events (less false positives) than the previous release, therefore, allowing a more reliable comparison between field measurements and DYNAMAP predictions [32].

**Figure 9:**
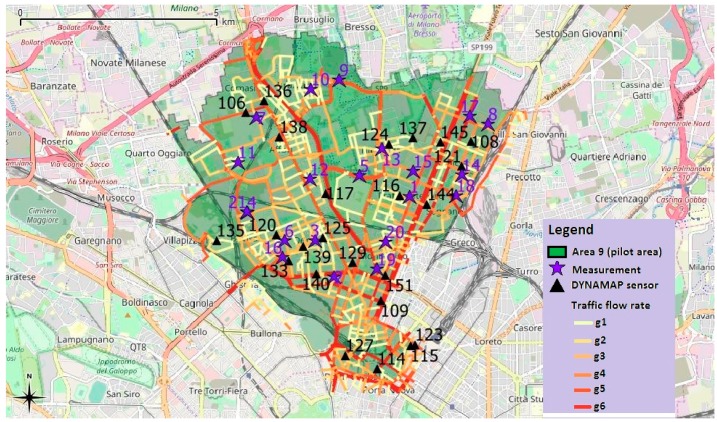
District 9 of the city of Milan city. Streets color corresponds to the different groups of streets according to range of non-acoustic parameter *x*: (0.0–3.0) (*g*_1_), (3.0–3.5) (*g*_2_), (3.5–3.9) (*g*_3_), (3.9–4.2) (*g*_4_), (4.2–4.5) (*g*_5_), (4.5–5.20) (*g*_6_). Black triangles and purple stars represent the sites where the monitoring stations are installed and the position of test measurements, respectively.

The test measurements were performed in 21 locations within District 9 (purple stars in Figure 9 and Table 3 for detailed addresses) equally distributed in the six groups of roads. The measurement sites were located at arbitrary points distributed within the pilot area of Milan and with different noise propagation conditions. In particular, sites were selected in order to test the system in complex scenarios where the noise from roads belonging to different groups may contribute. Special attention was given to avoid non-traffic noise sources such as technical systems (thermal power stations or ventilation systems), construction sites, railway, and tram lines, interfering with the measurements. Figure 9 also contains the position of the 24 monitoring stations together with the indication of the six groups of roads represented by different colors.

### 2.7. DYNAMAP Sensors Calibration

**Figure 10:**
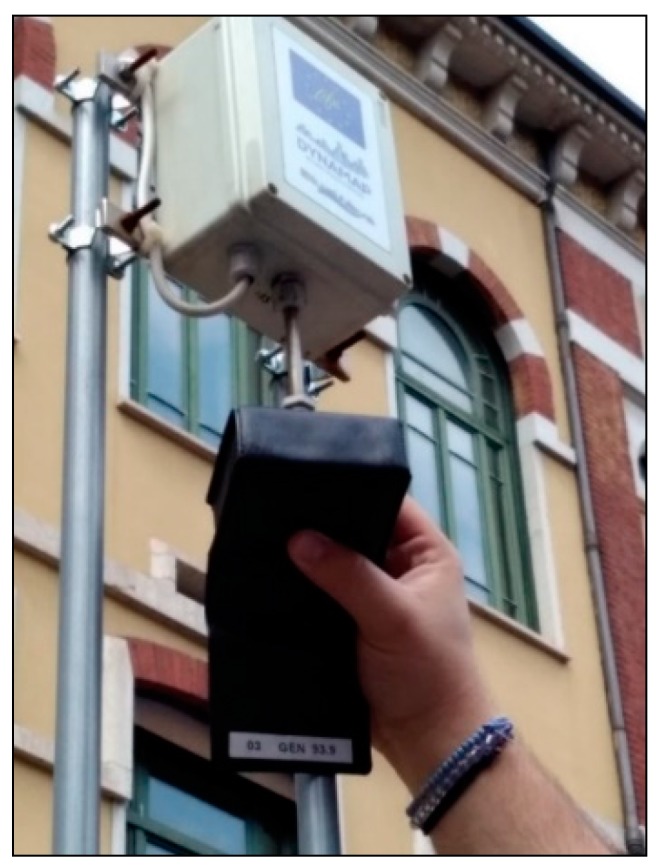
Operation of calibration on DYNAMAP sensor.

The correct assessment of DYNAMAP operation needs a careful evaluation of noise sensor network. The first evaluation activity involved DYNAMAP sensors calibration. The sensors have a characteristic accuracy which needed to be verified prior to their use. To this end, a field calibration procedure has been implemented with the help of a Class 1 calibrator (emission level 94 dB at 1 kHz, see Figure 10). The deviations of DYNAMAP sensors with respect to the calibrator are reported in Table 4. This value has been employed to correct the noise levels recorded by the corresponding noise sensor. In Table 4, the label N.C. (Not Calibrated), referred to three monitoring stations and means that these sensors could not be on-site calibrated by the operator because of safety reasons.

### 2.8. DYNAMAP Sensors Reliability

**Figure 11:**
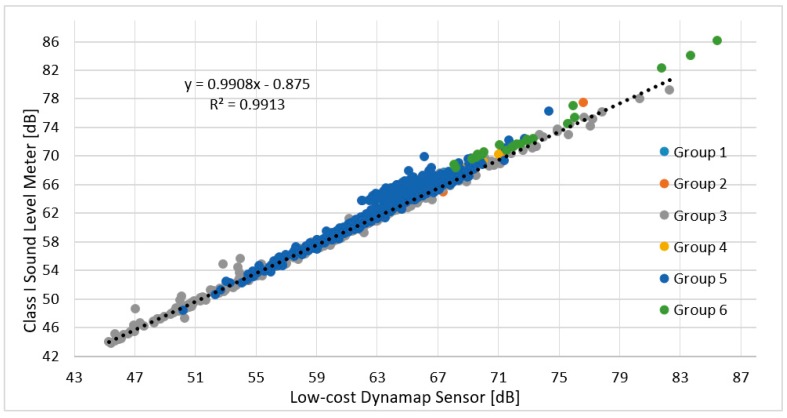
Correlation between the Class 1 Sound Level Meter and DYNAMAP Sensors. Different colors refer to sensors in each group of streets.

The second evaluation activity aimed at verifying the reliability of DYNAMAP sensors by comparing their readouts with a Class 1 sound level meter. The sound levels measurements (10 short duration measurements (≈1 h) and 2 measurements of 24 h) were performed on 12 monitoring sites (two sites for each group of roads), placing the microphone in the same position of the DYNAMAP sensor. The results of the tests expressed in *Leq^τ^* *_s_* with τ = 5 min, are summarized in Figure 11, showing the correlation between Class 1 sound level meter and DYNAMAP sensor. We obtained a high correlation (R^2^ = 0.99) with a mean deviation between the two sets of measurements of 1.0 ± 0.9 dB.

## 3. Results

In this section, we will describe the major steps to obtain an overall assessment of the project in terms of accuracy and reliability. A preliminary investigation [45] showed that the system is affected by different sources of error whose origin must be taken into account to minimize and eventually correct them. In the following, we provide a description of the measurement campaign and of the accuracy of both traffic model and DYNAMAP prediction.

### 3.1. Traffic Flow Data

In order to assess the validity of the traffic flow model, used to describe the non-acoustic parameter *x*, we performed a series of measurements of both traffic flow and noise at randomly selected sites and in correspondence of the noise monitoring stations, and compared them with the traffic model database. This test is important because the parameter *x* determines the group membership and therefore its dynamic behavior. In case the traffic model prediction is not accurate enough, DYNAMAP prediction could be sensibly affected.

**Figure 12:**
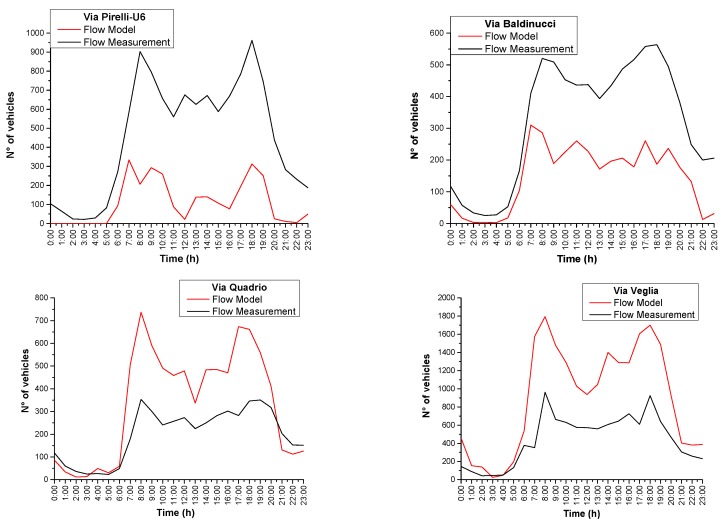
Comparison between hourly traffic flow (number of vehicles per hour) and traffic flow model calculations. Here, Via Pirelli-U6 (*g*_2_), Via Baldinucci (*g*_2_), Via Quadrio (*g*_4_), Via Veglia (*g*_5_) are some selected locations corresponding to the position of monitoring stations.

As one can see from Figure 12, there are significant differences between the traffic flow model predictions and measurements. Possible causes can be found in changes of traffic conditions (the model refers to a 2012 road network) and the incapability of the model to manage traffic conditions characterized by low flows (it has been designed and calibrated to deal with critical traffic situations). Consequently, in some cases the “real” total daily vehicle flow can significantly differ from the one attributed to a specific road using the flow model. This may result in jumps of group membership and, therefore, inaccurate predictions.

In Table 5, we report the comparison between the results of total traffic flows, in the form of the non-acoustic parameter *x*, obtained from the model calculations and the recent measurements in the same sites. Differences, or group jumps, occurred in particular for the case of Via Pirelli which became a congested road in recent years (from *g*_2_ to *g*_4_). As is apparent from Table 5, deviations of the predicted values *x* are within about 10% for groups *g*_3_–*g*_5_, and much higher for other groups.

### 3.2. DYNAMAP Predictions

In the following, we report the comparison between traffic noise measurements with the corresponding DYNAMAP predictions, $Leq_{s}^{\tau }\left(t\right)$, with *t* = (5, 15, 60) min. The different updating time intervals correspond to the three time-periods within the 24 h of a day: *t* = 5 min (07:00–21:00), *t* = 15 min (21:00–01:00), and *t* = 60 min (01:00–07:00). The DYNAMAP prediction of $Leq_{s}^{\tau }\left(t\right)$ at a site *s* within the network can be obtained from the relation reported in Equation (11). The reference values for the 21 selected sites *Leq_ref g,s_* that is the “static” level contribution from different groups are reported in Table 6. They illustrate how different groups contribute to the local noise level. The major contribution to the local site level, *Leq_ref g,s_*, in general, comes from the group *g* the site belongs to (see bold figures in Table 6). For example, for Site 1, which belongs to group *g*_5_, the most significant contribution comes from *Leq(s)_ref(g_*_5*)*_. However, each local site level is subject to the influence of nearby streets through other groups, as is apparent from Table 6. In particular, roads characterized by low traffic flow generally are mostly influenced by neighboring higher flow roads (see as an example Site 10, 18, and 20 of group *g*_1_).

**Figure 13:**
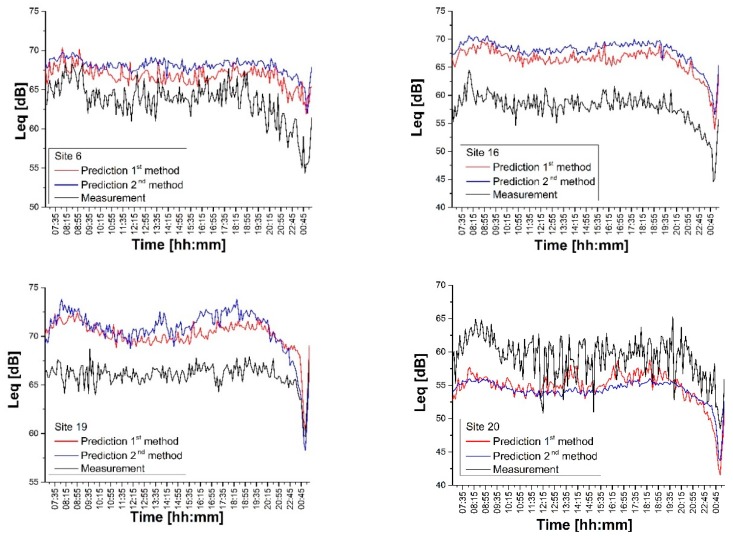
Comparison between traffic noise measurements at Sites 6, 16, 19, 20 and the corresponding DYNAMAP predictions according to two calculation methods (cfr. Equation (8) or Equation (10)).

The comparison between traffic noise measurements, $Leq_{s}^{}{\left(t\right)}_{m}$ and DYNAMAP predictions, $Leq_{s}^{}\left(t\right)$ at site *s* is based on the evaluation of the mean deviation:

(12) $$<\varepsilon_{Leq_{s}}>\;=\frac{1}{N}\overset{N}{\underset{k=1}{\sum }}\left|Leq_{s}^{}\left(k\right)-Leq_{s}^{}{\left(k\right)}_{m}\right|$$

where the summation index *k* extends over three time periods (24 h-*N_Tot_* = 190; day 07:00–21:00 h-*N*_5*min*_ = 168; evening 21:00–01:00 h-*N*_15*min*_ = 16; night 01:00–07:00 h-*N*_1*h*_ = 6). The results of the comparison between measurements and predictions (cfr. Equation (11)) according to the two calculation methods (cfr. Equation (8) or Equation (10)) are reported in Figure 13 for a representative number of sites (Sites 6, 16, 19, 20).

Figure 13 shows how both methods provide predictions with similar trends and deviations. Both methods are affected by a systematic, almost constant, error, most likely introduced by the traffic flow model (see discussion below). The latter should have a higher influence on the second prediction method as it takes on the contribution of all noise monitoring stations (see Equation (10)). However, the second method should be more robust in case one or more noise monitoring are offline. Site 20 presents higher discrepancies with high intermittency patterns especially during the day-time due to both the small integration time (5 min) and the irregular traffic flows in local roads.

In Table 7, we report the total daily mean deviation (24 h) for the two prediction methods in all the 21 test measurements.

## 4. Discussion

In the following, we will discuss a possible solution to improve DYNAMAP prediction within a reasonable range of error. For simplicity, we will consider 1h as updating time scale and the first prediction method based on Equations (8) and (11) for the calculation of the mean variation of each group, $\delta_{g}^{\tau }\left(t\right)$ and presented in Section 2.3.

### Prediction Corrections

A number of selected sites have been chosen to compare the results of field measurements with the corresponding DYNAMAP predictions.

**Figure 14:**
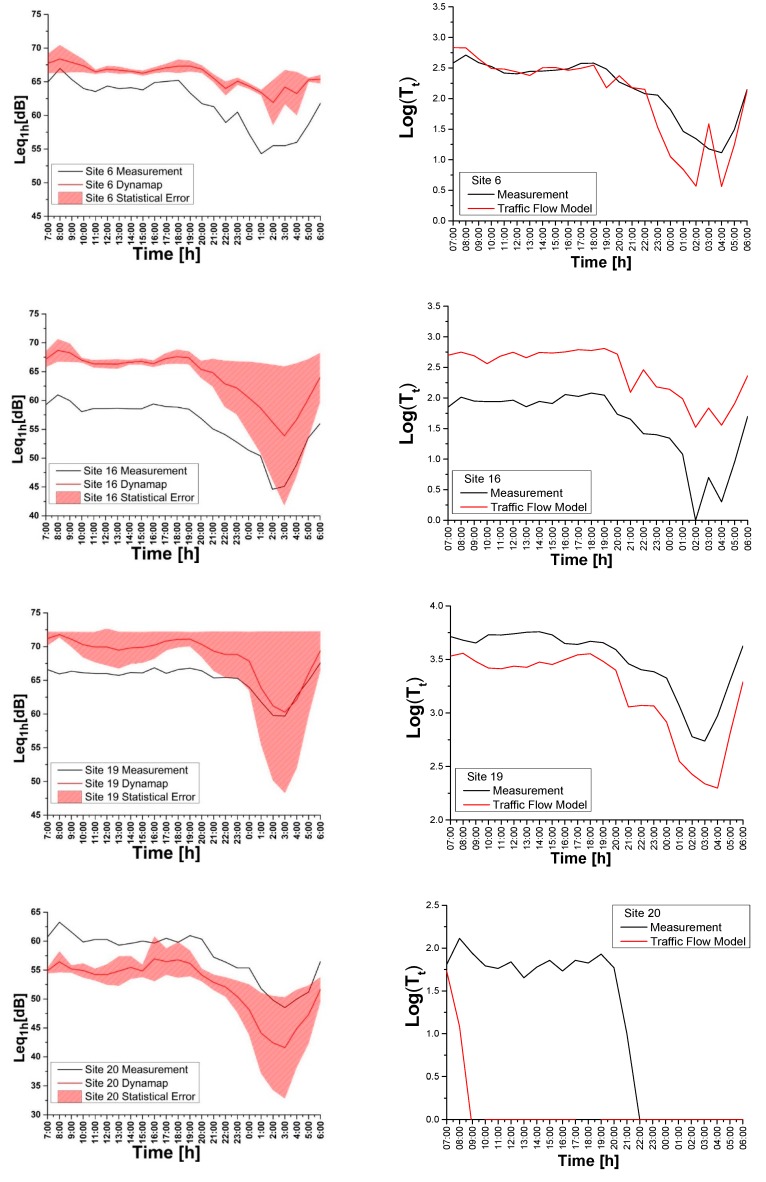
(Left side) Comparison between traffic noise measurements and DYNAMAP predictions at: Site 6 (group *g* = 3), Site 16 (group *g* = 4), Site 19 (group *g* = 6), Site 20 (group *g* = 1). The colored band represents the 1σ confidence level. (Right side) Comparison between traffic flow measurements and AMAT traffic model at the same sites.

Figure 14 (left part) presents a relevant discrepancy between predictions and measurements, which can be higher during the daytime. Each figure shows the error bands obtained from the propagation error associated with the variability of $\delta_{g}^{\tau }\;\left(t\right)$ within each group *g.* During the day time (07:00–21:00) the mean group discrepancy remains within 1 dB, whereas in the evening-night time (21:00–07:00) the high “volatility” of traffic noise pushes it to about (2–4) dB.

The almost constant gap between measurements and predictions in different period of the day suggested us to search for a systematic error inherent the DYNAMAP calculation method; systematic error which is most likely correlated to the vehicular flow employed in the prediction model. In fact, $\delta_{g}^{\tau }\left(t\right)$ is calculated with respect to *Leq_ref g_*, obtained from CADNA software using as input information on the number of vehicles/hour at the reference hour (8:00–9:00).

During the measurement campaign, we simultaneously recorded the traffic flows. This allowed us to compare the logarithm of traffic flow measurements with the traffic flow model calculations for Sites 6 (*g*_3_), 16 (*g*_4_), 19 (*g*_6_), and 20 (*g*_1_) as illustrated in Figure 14 (right part), respectively. The traffic flow data have been provided by Agenzia Mobilità Ambiente Territorio (AMAT), the agency in charge of the traffic mobility at the City Hall [46]. In the described examples, the model yields more reliable results for highly traffic roads belonging to groups *g*_3_, *g*_4_, and *g*_6_, than for lower flow roads as in *g*_1_, as already reported in a previous preliminary work [45].

As it is apparent from Figure 14, there is a gap between the prediction and the measurements of $Leq_{s}^{\tau }\left(t\right)$. The observed constant shift might be the result of inaccuracies of the traffic model in describing the traffic flow, especially for low traffic roads. Such shift is regarded as a systematic error.

To quantify this discrepancy and try to correct it, we calculate for each site the relative mean deviation (*ε_L_*) between hourly traffic noise measurement level, $Leq_{s}{\left(1h\right)}_{m}$, and the corresponding hourly DYNAMAP prediction level, $Leq_{s}{\left(1h\right)}_{}$, over the day and night period, defined as

(13) $$\varepsilon_{L}=\frac{1}{N}\overset{N}{\underset{k=1}{\sum }}\frac{\left(Leq_{s}{\left(k\right)}_{m}-Leq_{s}{\left(k\right)}_{}\right)}{Leq_{s}{\left(k\right)}_{m}}$$

where the summation index *k* extends over two time zones (day 07:00–21:00 h → *N*_1*h*_ = 14; evening-night 21:00–07:00 h → *N_1h_* = 10). The relative error is then averaged over all roads belonging to the same group, in order to represent the average hourly values of the road group ($\bar{\varepsilon_{L}}$). Furthermore, we consider the relative deviation (*ε_F_*) between measurement and model for the logarithm of the traffic flow at the reference time, *Log F*_(8:00–9:00)_,

(14) $$\varepsilon_{F}=\frac{Log\left(F_{\left(8:00-9:00\right)Meas.}\right)-Log\left(F_{\left(8:00-9:00\right)Model}\right)}{Log\left(F_{\left(8:00-9:00\right)Meas.}\right)}$$

where *Log*(*F*_(8:00–9:00)*Model*_) is the logarithm of the flows from 8:00 to 9:00 of the 2012 traffic model. Then we calculate the mean deviation of all sites belonging to the same group, $\bar{\varepsilon_{F}}$.

**Figure 15:**
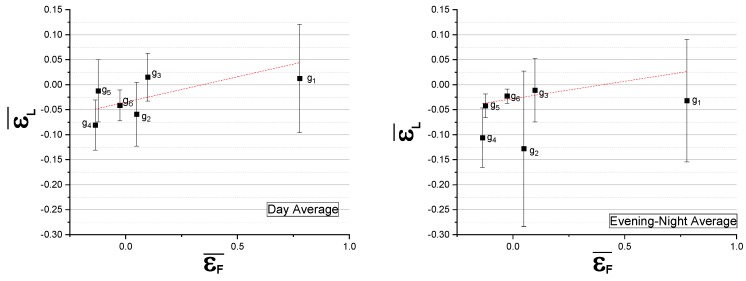
Relative mean hourly deviation between traffic noise measurements and the corresponding DYNAMAP predictions $\bar{\varepsilon_{F}}$ vs. the relative deviation between the logarithm of traffic flow measurements and the corresponding model calculations at the reference hour (8:00–9:00) $\bar{\varepsilon_{F}}$ for each group separately. The results refer to: Day time (07:00–21:00) (Left panel), and Evening-Night time (21:00–07:00) (Right panel) periods. The dashed line is just a guide for the eye.

These values for $\bar{\varepsilon_{L}}$ and $\bar{\varepsilon_{F}}$ are plotted in Figure 15, illustrating, to some degree, a relationship between traffic flow deviations and noise level errors. This relationship will be treated as a systematic error and taken into account within the DYNAMAP scheme.

**Figure 16:**
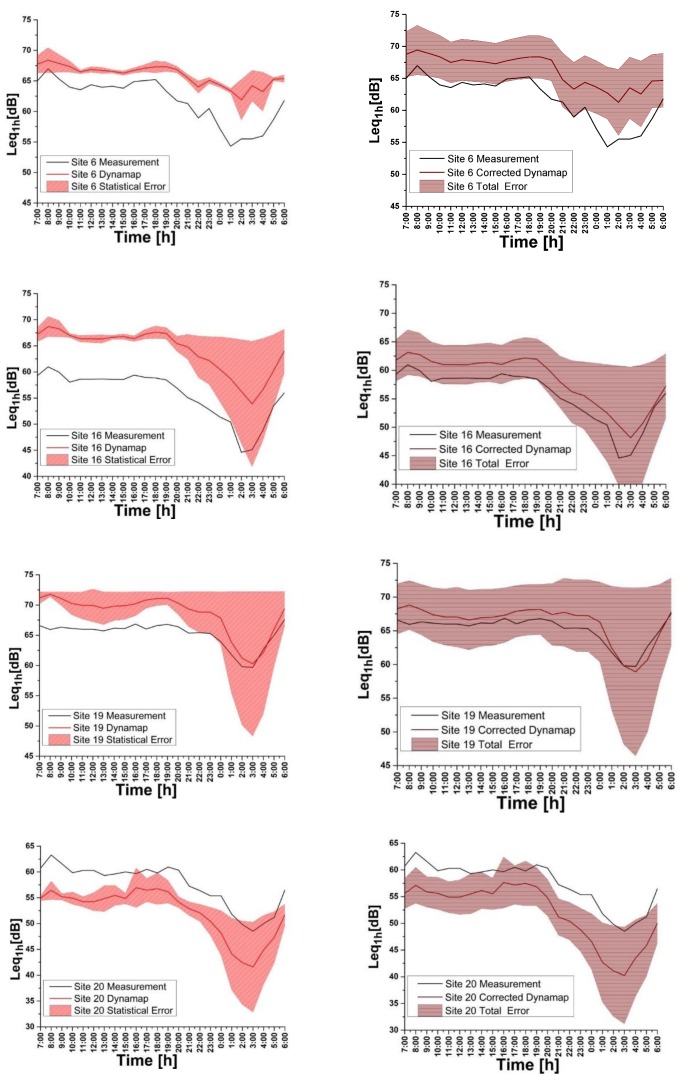
(Left part) Comparison of traffic noise measurements and DYNAMAP (non-corrected) prediction for: Site 16 (Upper panel), Site 19 (Middle panel), Site 20 (Lower panel). (Right part) Comparison of traffic noise measurements and DYNAMAP (corrected) prediction for the same sites. In the figure, the total error is displayed.

We thus obtain the corrected hourly value for the predicted noise level (Leq(1h)), by multiplying the different hourly values of the predicted noise level times the relative mean group deviation, expressed in percentage terms [1 + $\bar{\varepsilon_{L}}$ (*g*)]. The results of this operation are shown in Figure 16 (Right part, red line). We observe a general improvement of the prediction for these sites. In the graphics, the uncertainty bands include both the statistical and systematic errors (total error).

In Table 8, we report both the site mean hourly non-corrected, *<ε_Leq_>_N_*, and corrected prediction errors, *<ε_Leq_>_C_*, for all measurement sites, obtained through the comparison between the hourly non-corrected or corrected prediction levels and the hourly measurement levels, as shown in Equation (12).

The correction yields better predictions in many cases, but in others it remains poor. A median-based correction, *<ε_Leq_>_M_*, is also reported in Table 8. This quantity is less sensitive to outliers and, consequently, it provides more realistic estimates of the corrections. Finally, the right column of Table 8 shows the group mean errors calculated by averaging over the roads belonging to each group. The highest discrepancies are found for group *g*_1_ as a consequence of the poor descriptive capabilities of the traffic flow model. Except for this, the results obtained for the group median-average error, *<ε_Leq_>_M_*, is below 3 dB.

**Figure 17:**
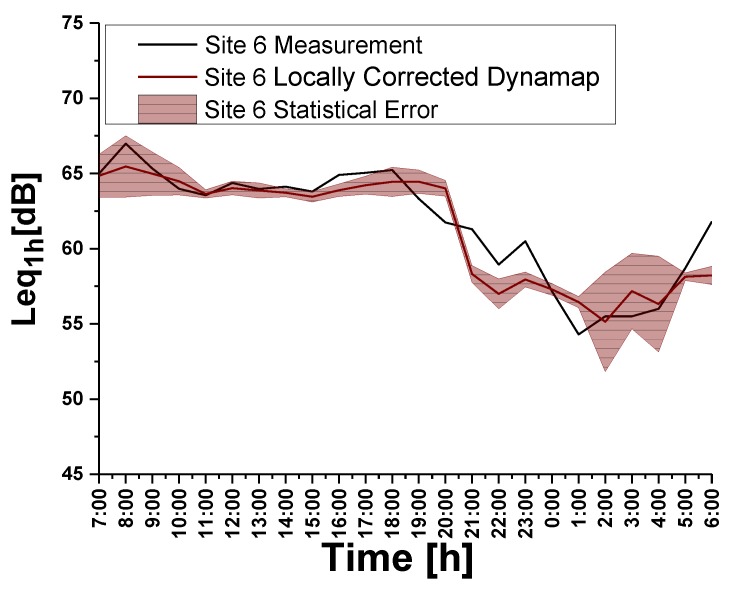
Comparison between traffic noise measurement and the “locally corrected” DYNAMAP prediction for Site 6.

Therefore, excluding group *g*_1_, for which a specific analysis needs to be developed, the prediction error of roads belonging to other groups, upon a systematic error correction *<ε_Leq_>*_C_, remains below 3 dB for each site, with the exception of Sites 6 (*g*_3_) and 7 (*g*_2_). The latter must be treated differently if we require that the 3 dB constrains must apply to all sites belonging to a group. We took 3 dB as a reference accuracy value as retrieved from the Good Practice Guide for strategic noise mapping [47]. As an example, consider site 6 (*g*_3_). Correcting the predicted noise level using its own relative traffic flow deviation (not the group mean), we obtain the results reported in Figure 17, that correspond to *<ε_Leq_>*_C_ = 1.1 dB.

**Figure 18:**
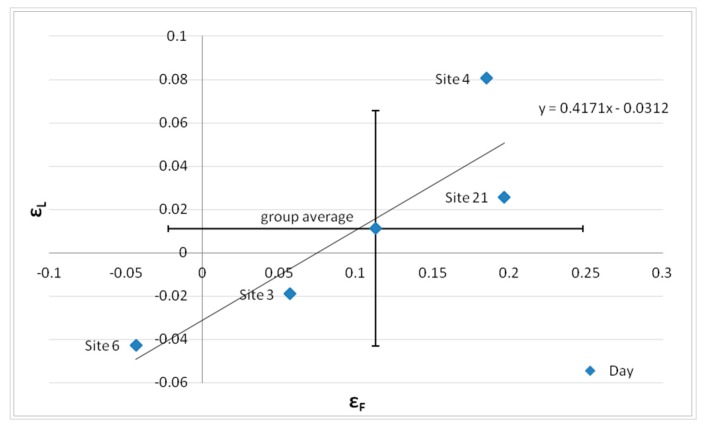
Relative mean hourly deviation between traffic noise measurements and the corresponding DYNAMAP predictions, *ε_L_*, versus the relative deviation between the logarithm of traffic flow measurements and the corresponding model calculations at the reference hour (8:00–9:00), *ε*_F_, for each site of group 3.

This result suggests that in order to get an effective correction, the relative error between the measured and the model traffic flow (8:00–9:00) in a given road stretch has to be bound within an interval that depends on the group it belongs to. In Figure 18, for example, we report the relative mean hourly deviation between traffic noise measurements and the corresponding DYNAMAP predictions, *ε_L_*, against the relative deviation between the logarithm of traffic flow measurements and the corresponding model calculations at the reference hour (8:00–9:00), *ε_F_*, for each site of group *g*_3_.

**Figure 19:**
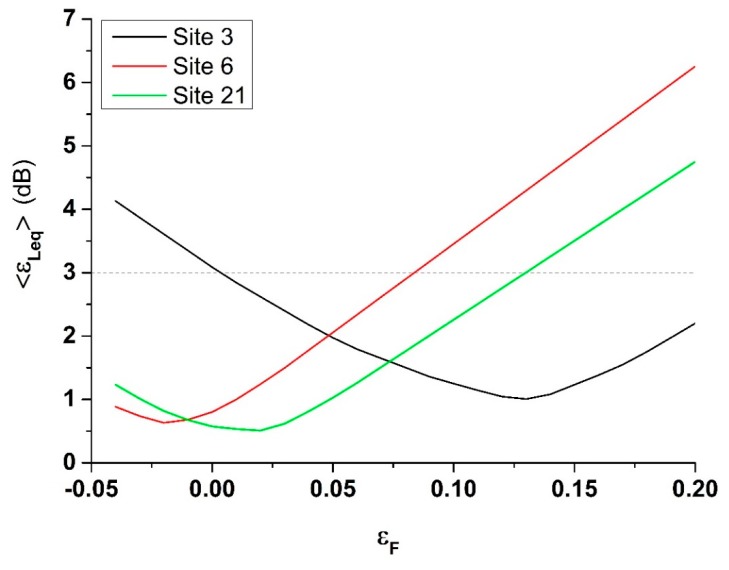
Mean prediction error <*ε_Leq_*> as function of the relative traffic flow error (8:00–9:00) *ε_F_* for Sites 3, 6, and 21. The graphs have been obtained assuming for simplicity that the relation between *ε_L_* and *ε_F_* is linear within group *g*_3_ (see Figure 18). The dashed line represents the 3 dB threshold.

Figure 18 has been obtained assuming for simplicity that the relation between *ε_L_* and *ε_F_* is linear within group *g*_3_. In this case, in order to get a prediction error <3 dB for each site, the relative error on the traffic flow can vary by about ±0.10 with respect to the minimum found for the single site, as it can be observed in Figure 19 for Sites 3, 6, and 21.

In Figure 19, the minimum prediction error is obtained near the corresponding site-specific flow error. It does not match exactly the value reported in Figure 18 because we are using a linear dependence between *ε_L_* and *ε_F_* (see Figure 18). In other words, the mean value of the relative error on the traffic flow of a given group *g*, $\bar{\varepsilon_{F}}$, (the one that has been used in the correction procedure of DYNAMAP prediction) must be bound within an interval that can be determined as follows: if we take $\bar{\varepsilon_{F}}$ centered at the minimum of the relative error of the site-specific traffic flow, *ε_F,S6m_* for the case of Site 6, it means that $\bar{\varepsilon_{F}}$ *= ε_F,S6m_* can have a maximum standard deviation σ = ±(0.10) to satisfy the condition about the mean prediction error, <*ε_Leq_*> < 3 dB. Therefore, $\bar{\varepsilon_{F}}$ must belong to an interval (*ε_F,S6m_* − 0.10, *ε_F,S6m_* + 0.10). This procedure has to be repeated for each site of the group. If these conditions are met, all sites will have <*ε_Leq_*> < 3 dB. This means that the traffic model must provide flow values for the streets belonging to each group with comparable accuracy in order that the error remains within the same threshold for all sites of the group.

As for roads characterized by low traffic flows, such as those belonging to group *g*_1_, the application of the correction based only on the relative deviation of the local traffic flow is not effective, because in these cases, the noise level is not mainly determined by the local traffic, but by that of busier nearby roads. In these cases, we may think to reassign them to other groups, applying the correction of the group whose contribution in the prediction of the noise level is predominant.

## 5. Conclusions

DYNAMAP is an automatic monitoring system, based on customized low-cost sensors and a software tool implemented in a general purpose GIS platform. It has been developed and built in two pilot areas located along the A90 motorway that surrounds the city of Rome (Italy) and inside the agglomeration of Milan (Italy). This paper describes the final assessment of DYNAMAP system implemented in the pilot area of Milan. The statistical-based nature of the project relies on the high degree of correlation between what we called as non-acoustic parameter (total traffic flow) and traffic noise levels. This correlation allowed an accurate description of the traffic noise due to clusters of roads (described as a single noise map) from the information recorded from a few monitoring stations distributed all over the pilot area.

The paper includes the description of two procedures for updating the acoustic maps: one based on the average of the noise recorded by the monitoring stations in each group (1st method) and the other based on a two-cluster expansion scheme performed directly over the noise recorded by the 24 monitoring sensors distributed over the six groups of roads (2nd method). Both methods provided similar results though the second one was more robust in the case where one or more noise monitoring stations went offline. This is because the lack of information from one sensor (or more than one) is not as disruptive as for the first method. Indeed, we will have a 25% of missing information (1st method) against 4% (2nd method) in case of missing data from one sensor. In order to validate the system, each monitoring station was calibrated and cross-checked with Class I sound meters. A field measurement campaign was performed in order to compare the results of noise measurements and traffic flow with the corresponding estimated values of the noise map and of the traffic model.

In terms of accuracy, the predictive capability of DYNAMAP was mainly associated with the related accuracy of the chosen non-acoustic parameter (traffic flow). For this reason, a poor accuracy of the non-acoustic parameter is directly reflected on the noise prediction error. A method to correct the predicted noise levels in an arbitrary location and, therefore, limit the overall mean error within 3 dB for all groups of roads was illustrated. However, the requirements to keep the prediction error within 3 dB for each site established a serious constraint on the traffic flow model accuracy. This means that a significant improvement would be obtained by implementing a more realistic traffic flow model. This would reduce the systematic error and, therefore, enhance the overall reliability of DYNAMAP prediction. Hopefully, the implementation of mobile sampling and, more generally, of participatory sensing both for noise and traffic data would help reduce the uncertainty of noise maps. Conversely, this result may cause either an incorrect evaluation of the exposed population or improper noise action plans. Therefore, the uncertainty analysis in the creation of noise maps is a fundamental key tool to design noise action plans on extended areas.

## Figures and Tables

**Table 1:** Monitoring sensor information: code, group membership, non-acoustic parameter, *x* = Log(T_T_), and cluster membership according to the performed analysis (12 sensors belong to Cluster 1 and 12 to Cluster 2).

Sensor Code	Group *g_i_*	*x* = Log(T_T_)	Cluster
135	1	2.89	2
137	1	1.90	2
139	1	1.13	2
144	1	2.94	2
108	2	3.06	1
124	2	3.50	2
125	2	2.69	2
145	2	3.42	2
115	3	3.58	2
116	3	3.60	2
120	3	3.74	1
133	3	3.75	2
121	4	4.06	1
127	4	3.90	2
129	4	3.94	1
138	4	4.19	2
106	5	3.90	1
123	5	4.30	1
136	5	4.21	1
151	5	4.40	1
109	6	4.75	1
114	6	4.58	1
117	6	4.85	1
140	6	4.70	1

**Table 2:** Mean values of $\bar{\beta }$_1_ and $\bar{\beta }$_2_ for the six groups of *x* = Log(T_T_) within District 9.

Range of *x*	0.0–3.0	3.0–3.5	3.5–3.9	3.9–4.2	4.2–4.5	4.5–5.2
$\bar{\beta }$_1_	0.99	0.81	0.63	0.50	0.41	0.16
$\bar{\beta }$_2_	0.01	0.19	0.37	0.50	0.59	0.84

**Table 3:** Location of noise monitoring stations and measurement sites (cfr. Figure 9). The group index of each sensor and site are indicated within parenthesis.

**Station (Group *g_i_*)**	**Address**	**Station (Group *g_i_*)**	**Address**
106 (5)	Via Modigliani	127 (5)	Via Quadrio
108 (2)	Via Pirelli	129 (4)	Via Crespi
109 (6)	Viale Stelvio	133 (3)	Via Maffucci
114 (6)	Via Melchiorre Gioia	135 (1)	Via Lambruschini
115 (3)	Via Fara	136 (5)	Via Comasina
116 (3)	Via Moncallieri	137 (1)	Via Maestri del Lavoro
117 (6)	Viale Fermi	138 (4)	Via Novaro
120 (3)	Via Baldinucci	139 (1)	Via Bruni
121 (4)	Via Pirelli	140 (6)	Viale Jenner
123 (5)	Via Galvani	144 (1)	Via D’intignano
124 (2)	Via Grivola	145 (2)	Via F.lli Grimm
125 (2)	Via Abba	151 (5)	Via Veglia
**Site (Group *g_i_*)**	**Address**	**Site (Group *g_i_*)**	**Address**
1 (5)	Via Suzzani	12 (2)	Via Pastro
2 (2)	Via Bernina	13 (4)	Via Bauer
3 (3)	Via Ciaia	14 (2)	Via Polvani
4 (3)	Via Cosenz	15 (4)	Via Gregorovius
5 (5)	Via Majorana	16 (4)	Via Catone
6 (3)	Via Maffucci	17 (6)	V.le Sarca
7 (2)	Via Ippocrate	18 (1)	Via Boschi Di Stefano
8 (3)	Via Chiese	19 (6)	Via Murat
9 (5)	Via Moro	20 (1)	Via Sarzana
10 (1)	Via Marchionni	21 (3)	Via Cosenz
11 (1)	Via Gabbro		

**Table 4:** Calibration deviations of DYNAMAP sensors (values are in dB; N.C.: Not Calibrated).

Sensor	Site	Deviation [dB]
145	Via F.lli Grimm	−0.1
136	Via Comasina	−0.5
138	Via Novaro	+0.2
125	Via Abba	−0.6
123	Via Galvani	−0.2
115	Via Fara	−0.1
114	Via Melchiorre Gioia	−0.8
127	Via Quadrio	N.C.
140	Viale Jenner	−0.9
133	Via Maffucci	−0.3
120	Via Baldinucci	−0.5
129	Via Crespi	−0.7
151	Via Veglia	−0.4
116	Via Moncalieri	−0.2
124	Via Grivola	−0.6
137	Via Maestri del Lavoro	−0.5
144	Via d’Intignano	−0.5
121	Via Pirelli	0.0
108	Via Pirelli	−0.2
135	Via Lambruschini	−0.1
109	Viale Stelvio	N.C.
106	Via Litta Modignani	+0.2
117	Viale Fermi	0.0
139	Via Bruni	N.C.

**Table 5:** Group assignment according to model calculations and flow measurements in correspondence to 10 noise monitoring stations.

Site (Street Name)	Group *g*_i_		Values of *x =* Log(T_T_)	
	Model	Meas.	Model	Meas.
Via Lambruschini	1	2	2.95	3.50
Via Maestri del Lavoro	1	2	2.90	3.41
Via Grivola	2	1	3.29	2.92
Via Pirelli	2	4	3.42	4.04
Via Fara	3	3	3.75	3.66
Via Baldinucci	3	3	3.54	3.89
Via Quadrio	4	3	3.90	3.68
Via Crespi	4	4	4.15	4.08
Via Comasina	5	5	4.33	4.25
Via Veglia	5	4	4.33	4.03

**Table 6:** Level contributions of each group, *Leq_refg,_*_s_ at 21 arbitrary chosen sites of District 9 (Figure 9). The group indices of each sites are shown in the second column. Bold figures represent the major contribution to the local site level.

Site	Group *g*_i_	*Leq_ref g_*_1*,s*_	*Leq_ref g_*_2*,s*_	*Leq_ref g_*_3*,s*_	*Leq_ref g_*_4,*s*_	*Leq_ref g_*_5*,s*_	*Leq_ref g_*_6*,s*_
**1**	**5**	21.1	47.8	56.8	28.3	**64.9**	37.7
**2**	**2**	12.0	**64.6**	15.0	15.0	15.0	59.6
**3**	**3**	0.0	56.1	**62.7**	0.0	0.0	0.0
**4**	**3**	17.5	25.3	**59.4**	48.8	51.9	0.0
**5**	**5**	29.7	25.9	32.4	29.4	**67.8**	33.6
**6**	**3**	41.3	45.9	**66.4**	34.5	27.0	28.0
**7**	**2**	24.1	**58.1**	51.4	17.8	42.2	45.6
**8**	**3**	21.1	21.9	**53.9**	49.4	26.9	29.9
**9**	**5**	8.1	32.5	35.2	43.6	**62.3**	0.0
**10**	**1**	38.2	**43.0**	27.2	25.2	32.7	28.4
**11**	**1**	**55.8**	20.6	32.0	37.7	42.9	0.0
**12**	**2**	41.1	**62.4**	24.5	20.8	48.8	40.2
**13**	**4**	42.1	56.0	38.3	**69.2**	41.9	38.8
**14**	**2**	44.3	**61.1**	51.9	45.6	36.0	34.4
**15**	**4**	12.5	29.7	29.8	**70.2**	50.5	33.2
**16**	**4**	33.2	30.6	47.9	**68.6**	54.3	37.1
**17**	**6**	25.4	24.0	34.4	51.3	50.6	**69.7**
**18**	**1**	49.0	45.0	**59.1**	56.9	57.0	53.3
**19**	**6**	24.2	32.6	39.7	38.3	37.3	**71.7**
**20**	**1**	51.0	38.7	**52.1**	30.6	35.6	36.1
**21**	**3**	17.0	15.8	**56.8**	48.2	51.7	0.0

**Table 7:** Summary of the total daily mean deviation (24 h) for the two prediction methods in all the 21 test measurements.

Site	Group *g*_i_	Mean Deviation–1st Method (24 h) [dB]	Mean Deviation–2nd Method (24 h) [dB]
10	1	6.4 ± 2.5	11.0 ± 2.6
11	1	3.2 ± 2.3	3.8 ± 2.1
18	1	6.5 ± 1.5	7.5 ± 1.4
20	1	4.7 ± 2.3	5.1 ± 2.3
7	2	1.7 ± 1.5	3.6 ± 1.4
12	2	7.5 ± 2.333	2.7 ± 2.0
14	2	3.4 ± 1.6	1.4 ± 0.9
3	3	2.8 ± 1.7	5.2 ± 2.0
4	3	3.0 ± 2.7	2.0 ± 1.9
6	3	3.2 ± 1.9	4.5 ± 2.0
8	3	2.0 ± 1.3	1.2 ± 1.1
21	3	1.7 ± 1.1	0.9 ± 0.7
13	4	4.0 ± 1.3	5.5 ± 1.4
15	4	3.0 ± 1.2	4.7 ± 1.0
16	4	8.4 ± 1.5	10.0 ± 1.3
1	5	4.9 ± 1.3	6.5 ± 1.5
5	5	2.0 ± 1.3	1.9 ± 1.4
9	5	1.3 ± 0.9	2.1 ± 1.2
17	6	1.4 ± 0.8	2.8 ± 1.1
19	6	4.0 ± 1.2	4.7 ± 1.6

**Table 8:** (Left part) Mean site prediction error without systematic error correction, <*ε_Leq_*>_N_, with systematic error correction, <ε*_Leq_*>_C_, and median average of the corrected prediction, <*ε_Leq_*>_M_. (Right part) Mean group non-corrected prediction error, <*ε_Leq(g_*_)_>_N_, mean group corrected error, <ε*_Leq_*_(g)_>_C_, and group median average, <*ε_Leq(g_*_)_>_M_. All values are in dB.

Site	Group *g*_i_	*<ε_Leq_>_N_*	*<ε_Leq_>_C_*	*<ε_Leq_>_M_*	Group *g_i_*	<ε*_Leq_*_(g)_>_N_	<ε*_Leq_*_(g)_>_C_	<ε*_Leq_*_(g)_>_M_
10	1	5.0	5.2	5.2	1	5.3	5.1	5.2
11	1	4.5	4.0	4.1	2	4.2	3.2	2.8
18	1	6.4	6.1	6.1	3	2.5	2.5	2.8
20	1	5.3	5.5	5.6	4	5.3	2.4	2.1
7	2	1.9	4.0	2.9	5	2.6	2.4	2.2
12	2	7.8	2.5	3.8	6	3.4	1.3	1.3
14	2	2.8	3.1	1.6				
3	3	1.8	2.3	2.5				
6	3	4.2	4.5	5.9				
4	3	2.1	1.5	2.0				
21	3	1.8	1.5	0.7				
13	4	4.1	2.0	0.8				
15	4	3.3	2.6	1.3				
16	4	8.4	2.6	4.2				
1	5	4.5	3.0	4.4				
5	5	1.9	2.4	1.2				
9	5	1.4	1.9	1.0				
19	6	3.4	1.3	1.3				

## Acronyms

In this section, we provide the list of acronyms employed throughout the manuscript:

AMAT	Agenzia Mobilità Ambiente Territorio
ANEs	Anomalous Noise Events
ANED	Anomalous Noise Events Detection
ARM	Advanced RISC Machines
CADNA	Computer Aided Noise Abatement
cfr	confer
CNOSSOS-EU	Common Noise Assessment Methods in Europe
DYNAMAP	DYNamic Acoustic MAPping
END	Environmental Noise Directive
e.g.,	exempli gratia
GIS	Geographic Information System
N.C.	Not Calibrated
RISC	Reduced Instruction Set Computer
